# Unusual Course of Splenic Marginal Zone Lymphoma

**DOI:** 10.4021/wjon700w

**Published:** 2013-09-27

**Authors:** Nay T. Tun, Kaihong Mi, John Smith

**Affiliations:** a Department of Medicine, Easton Hospital, USA

**Keywords:** Splenic marginal zone lymphoma, Rituximab, Bendamustine

## Abstract

A 53-year-old woman was diagnosed with splenic marginal zone lymphoma by pathological examination on left submandibular lymph node and bone marrow biopsies and markedly enlarged spleen. Four cycles of Rituximab chemotherapy were given. Seven months after finishing Rituximab chemotherapy, she developed left upper extremity swelling without evidence of deep venous thrombosis. Repeat PET/CT scan demonstrated multiple left axillary lymph nodes extending to left retroclavicular region and a new lymph node posterior to the left scapula. Biopsy of the lymph node demonstrated marginal zone lymhoma pattern with increased numbers of large cells, but not outright diffuse large B-cell lymphoma. Despite resuming rituximab, patient had persistent leukocytosis and severe anemia. Restaging PET/CT showed 3 new left anterior cervical lymph nodes and 1 new right axillary lymph node. Spleen has further enlarged. R-CHOP chemotherapy was started, which improved leukocytosis.After 4 cycles of R-CHOP, PET/CT showed new metabolic activity within right inguinal and abdominal lymph nodes. Patient was given one cycle of Bendamustine. She developed a possible “hematoma” in right medial elbow. However, MRI study revealed a subcutaneous deposit of the lymphoma. Patient needs consistently blood transfusion and she deteriorated quickly. Our patient had an aggressive course of splenic marginal zone lymphoma, not responding to four trials of chemotherapy although SMZL is well-known to be an indolent low grade lymphoma. This case report emphasizes the importance to individualize the treatment in SMZL patients and repeat bone marrow biopsy if the disease recurs.

## Introduction

Splenic Marginal Zone Lymphoma (SMZL) is an extremely rare low-grade B-cell lymphoma, comprising less than 2% of all non-Hodgkin lymphoma [1, 2]. This condition is one of the variants of marginal zone lymphomas where lymphoid tumors originate from B lymphocytes. Marginal zone in spleen is the most external layer of white pulp in spleen, where B cells predominate. The median age of occurrence in SMZL is 65 years while the median survival has been recorded to be approximately 10.5 years.

The pathogenesis of SMZL still remains unclear. Some data suggested that the malignant cells appear to have undergone somatic hypermutation, implying the presence of previous antigenic exposure. Recent studies confirmed the particular cytogenetic profile of SMZL from other B-cell lymphomas. The predominant aberration is 7q deletion [3-6] followed by gains of 3q, 5q, 9q, 12q and 20q, deletion 7q and 17p [4]. A European multicentric retrospective analysis confirmed the strong biased usage of Immunoglobulin V_H_ segments [5]. Clinical and epidemiological studies have identified an association between SMZL and infection with hepatitis C virus [7-10] which is likely due to HCV virus significantly downregulating tumor suppressive genes [11].

We present a patient with splenic marginal zone lymphoma, who failed Rituxan, R-CHOP and Bendamustine. She had an aggressive course of splenic marginal zone lymphoma with no evidence of transformation to high grade B-cell lymphoma.

## Case Report

A 53-year-old woman was admitted to the hospital because of abdominal pain, fatigue and anorexia for 2 days. The patient was in her usual state of health until approximately 2 days prior to the admission, when fatigue and anorexia developed, followed by abdominal pain. She reported a low grade fever, night sweats, and about 11pounds weight loss over the past year. She had some shortness of breath, but no cough.

Splenomegaly with multiple splenic infarcts had been diagnosed by CT scan approximately a year earlier after an episode of left upper quadrant pain developed and had been treated by Coumadin for 3 months. Transesophageal echocardipgram and extensive coagulation studies did not reveal any abnormality. The etiology for the splenomegaly was unclear at that time. CT scan done 5 months ago demonstrated a new splenic infarct. The patient resumed Coumadin. The patient had hypothyroidism and hyperlipidemia. She was a middle school teacher, never smoked, and did not use alcohol or illicit drugs. She did not have any recent history of travel or pets exposure. Her mother had coronary artery disease. Her father had diabetes mellitus. Her brother had hypothyroidism. Medications included Synthroid, Coumadin, and Rosuvastatin.

**Figure 1:**
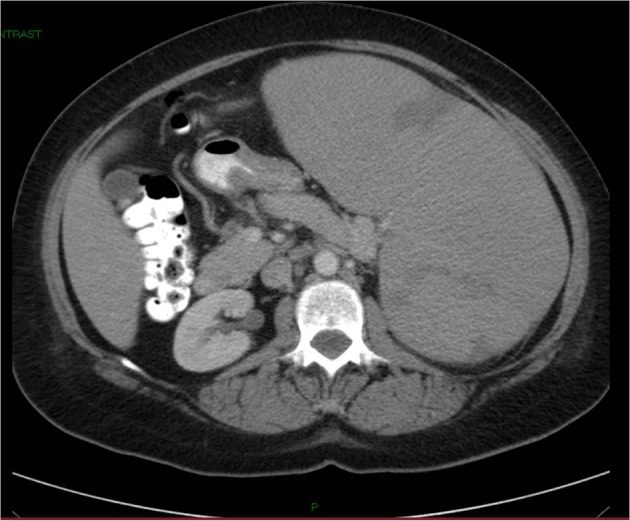
CT of abdomen and pelvis without contrast showing massive splenomegaly measuring 22.8 cm.

On examination, the patient appeared anxious. Her temperature was 36.2 °C, blood pressure 108/59 mmHg, pulse rate 97 beats per minute, respiration rate 14 breaths per minutes, and oxygen saturation 97% while she was breathing ambient air. There was no scleral icterus. The abdomen was soft, with tenderness in the epigastrium. The spleen was firm and nontender and extended to the left lower quadrant and the supraumbilical region. There were several palpable lymph nodes in the left neck with the largest one in the left submandibular region. The remainder of the examination was normal. CT scan of the abdomen, pelvic, and thorax with oral and intravenous contrast after admission showed multiple small or borderline enlarged lymph nodes in the left axilla, mediastinum, and retroperitoneum. The spleen was markedly enlarged 22.8 cm in length with peripheral wedge-shaped hypodensities, suggesting infarcts (Fig. 1). A test for human immnunodeficiency virus was negative. Epstein Barr Virus antibodies, cytomegalovirus antibodies and toxoplasma antibodies were negative. Direct antiglobulin test and test for cold agglutinins were negative. Electrocardiogram was normal. Serum protein electrophoresis did not reveal an Ig M kappa paraprotein. Tests for hepatitis B virus surface antibody and antigen and hepatitis C virus antibody were negative.

**Figure 2:**
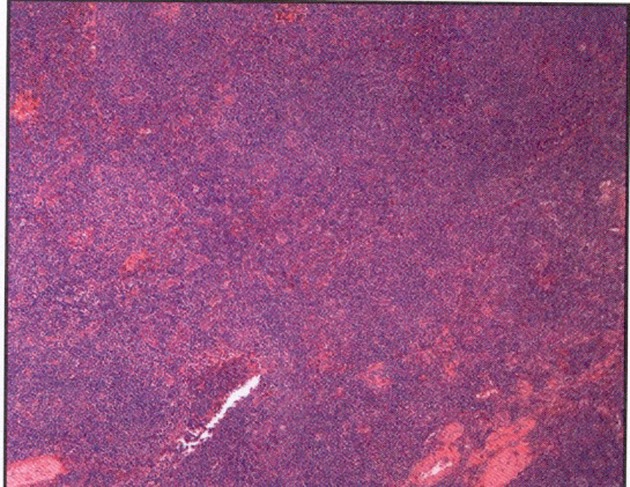
Left submandibular lymph node biopsy revealed a monotonous nodular infiltrate of small to medium sized lymphocytes with irregular nuclear contours with noted follicular colonization.

The left submandibular lymph node excisional biopsy and bone marrow biopsy were done. Pathological examination of the biopsy specimen of the lymph node revealed monotonous nodular infiltrate of small to medium sized lymphocytes with irregular nuclear contours, condensed chromatin, inconspicuous nucleoli and small to moderate amounts of cytoplasm, with noted follicular colonization (Fig. 2). Flow cytometry showed an expanded population of kappa-restricted CD19+ CD20+ CD23+CD5-CD10- B cells with unquantified CD5+CD10+ T cells. FISH analysis was negative for Ig H/BCL1 translocation, IgH/BCL2 translocation and for a MALT1 gene rearrangement. Pathological examination of the bone marrow revealed that hypercellular marrow (80%), and lymphoid aggregates that made up approximately 20% of the marrow cellularity and interstitial lymphoid aggregates. Flow cytometry performed on the bone marrow aspirate demonstrated an expanded population (10%) of kappa-restricted B cells that expressed CD19, CD20, CD45, HLA-DR but not CD5, CD10, CD103, CD11c. FISH analysis performed on bone marrow was positive for IgH rearrangement, while negative for IgH/BCL1 and IgH/BCL2 translocations and for ALK1 and BCL6 rearrangements.

Splenic marginal zone B-cell lymphoma with involvement of lymph node and bone marrow was diagnosed. Position Emission Tomography (PET) scan for staging after the bone marrow biopsy showed hypermetabolic activity was limited to the spleen and goiter, which was consistent with the history of thyroiditis. The grossly enlarged spleen had nearly homogenous increased metabolic activity, measuring at least 22 cm in cross-section with extension of the spleen below the iliac crest. There were some peripheral wedge-shaped areas of decreased activity, reflecting areas of splenic infarction. After obtaining the second opinion from physicians in University of Pennsylvania, four cycles of Rituxan chemotherapy were given. The patient tolerated the chemotherapy well.

There was no clinical evidence of recurrence of the lymphoma and no new lymphadenopathy until 9 months after initial diagnosis. She presented to the oncology clinic with left upper extremity swelling. Deep venous thrombosis work-up was negative. Repeated PET scan demonstrated multiple left axillary lymph nodes extending to left retroclavicular region and a new lymph node posterior to the left scapula. The spleen was 4 cm smaller compared to the previous PET scan. The wedge shaped defect in the spleen had resolved. Excisional biopsy of the left axillary lymph node was done. Pathology examination demonstrated marginal zone B-cell lymphoma with follicular colonization pattern and increased numbers of large cells. The histological findings were not those of outright diffuse large B-cell lymphoma. Decision was made to resume Rituxan.

On follow-up after finishing second round of 4 cycles of Rituxan, the white cell count was elevated at 148 × 10^9^/L with 95% lymphocytosis. Restaging PET scan showed 3 new left anterior cervical lymph nodes, about 8 mm in diameters in each of them. One new 2.5 cm lymph node was found in the right axilla. The spleen had enlarged compared with the PET scan 4 months ago. New areas of hypodensities within the lateral periphery of the spleen were consistent with new small infarcts. R-CHOP (Rituxan- Cyclophosphamide, Hydroxydaunorubicin, Oncovin, Prednisone) chemotherapy regimen was started after a port-A- catheter was placed. White cell count significantly decreased after 1st cycle of R-CHOP from 144 × 10^9^/L to 61.2 × 10^9^/L.

**Figure 3:**
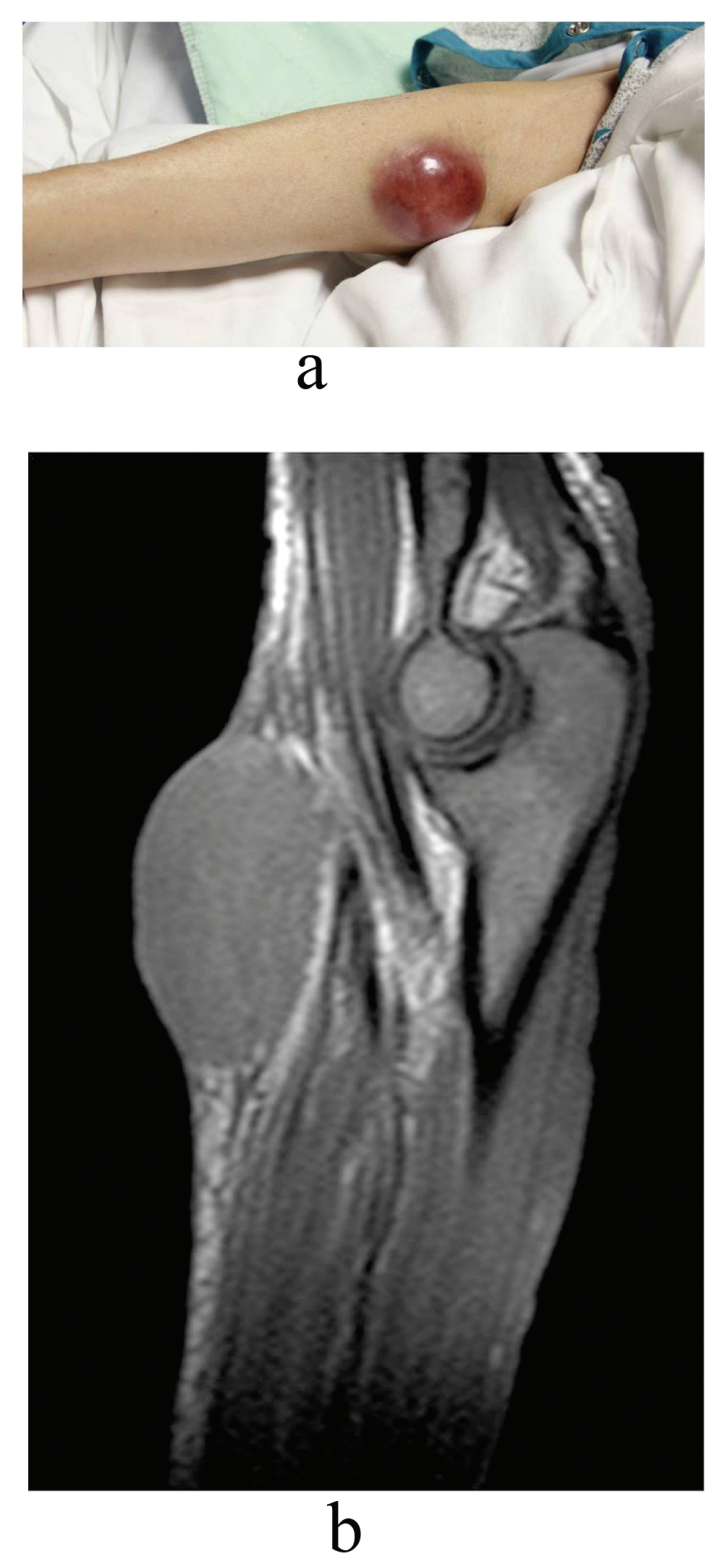
a). Right elbow suggestive of possible “hematoma” and b) MRI of right elbow suggested deposit of lymphoma.

After two cycles of R-CHOP, patient developed more fatigue, right facial numbness and right ptosis, being diagnosed with idiopathic right-sided Bell’s palsy. PET scan after 4 cycles of R-CHOP demonstrated interval resolution of previously described metabolically active cervical and axillary lymphadenopathy. New metabolic activity within right inguinal lymph nodes and abdominal lymphadenopathy were found with stable splenomegaly. Patient received 1 cycle of Bendamustine (Treanda). She developed a possible “hematoma” on right medial elbow (Fig. 3a). MRI of the right upper extremity without contrast revealed the mass was not of vascular origin, likely representing a deposit of the lymphoma (Fig. 3b). During this hospitalization, patient needs blood transfusion consistently and she deteriorated quickly. Decision was made by family for comfort measures.

## Discussion

Although the differential diagnosis for splenomegaly is broad, diseases that cause massive splenomegaly below the iliac crest are limited [12]. This patient had no known family history of the Gaucher’s disease and did not have hepatomegaly or bone disease. Most cases of SMZL do not require splenectomy for the diagnosis, which can be accurately determined on the basis of clinical splenomegaly, lymphocyte morphology, immunophenotype, cytogenetic analysis, and bone marrow histology. The diagnosis of splenic marginal zone lymphoma in this patient was based on the morphological and immunophenotypical studies of the excised left submandibular lymph node. Subsequent studies from bone marrow biopsy further confirmed the group of B cells with the same characteristics. Although nearly all patients have bone marrow involvement, unlike most other Non Hodgkin Lymphomas, lymphadenopathy is uncommon in SMZL [13].

The other features in our patient were recurrent splenic infarction, progressive and disseminated disease course without evidence of histological transformation to high grade NHL, and B symptoms on initial presentation, which is uncommon in SMZL. The B symptoms usually suggest for the poorer outcome [14]. One third of the SMZL patients have a serum monoclonal paraprotein (M-component), mostly IgM kappa, which is responsible for hypercoagulation in these SMZL patients. However, Ig M paraprotein was within normal range in this patient. The recurrent multiple infarcts were only observed in spleen, which was most likely caused by tumor infarcts. Our patient had a progressive disease course and she expired in one and half years after diagnosis of SMZL in spite of rituximab, R-CHOP, and Treanda treatments. The disease also disseminated to the subcutaneous tissue as manifested by tumor in right medial elbow. The recurrence of the lymphoma 7 months after rituximab treatment was evidenced by new active lymph nodes on PET scan. The pathologic studies of the excised left axillary lymph node confirmed the recurrence of marginal zone lymphoma with no evidence of outright diffuse large B-cell lymphoma although increased number of large neoplastic lymphoid cells were observed. Unfortunately, the bone marrow biopsy was not repeated in this case. High-grade transformation may occur in the bone marrow in this case, which is frequently refractory to therapy and associated with poorer outcome compared to the lymph node transformation [15].

Because of the high frequency of bone marrow involvement, about 95% cases are classified as Ann Arbor stage IV. It commonly follows an indolent course exceeding a median 10-year survival. However, in a minority of cases, it can pursue a more aggressive course with the possibility of transformation into a diffuse large B-cell lymphoma [15]. Poor prognostic factors include anemia, hypoalbuminemia, and an elevated lactate dehydrogenase level (LDH) [16]. Other adverse prognostic features include leukocyte count > 20,000 × 10^9^/L, lymphocytosis > 9,000 × 10^9^/L, presence of paraprotein, and an elevated β2-microglobulin levels [17-19]. In our patient, she had anemia and elevated LDH on initial diagnosis. When the disease relapsed, she had elevated β2-microglobulin, hypoalbuminemia, and severe lymphocytosis (white cell count 144.4 × 10^9^/L and 95% lymphocyte). She also had severe anemia, requiring multiple blood transfusions. Subcutaneous tissue was involved subsequently as well.

The goal of treatment in SMZL is to prolong survival and quality of life, manage symptoms, and prevent complications. Like other indolent Non Hodgkin lymphomas, “wait-watch” strategy is also applied to SMZL. About one third of patients do not require treatment initially. Treatment was indicated in patients with symptomatic splenomegaly, cytopenias, systemic symptoms, and autoimmune complications. Our patient did not have evidence of HCV infection, the treatment specifically to HCV with interferon and ribavirin would not be expected to produce a response [20, 21]. In the pre-rituximab era, splenic irradiation as a treatment option was reported in few cases and alkylating agent therapy, either alone or in combination CVP (cyclophosphamide, vincristine, prednisone) or CVP-R (CVP with rituxan) or R-CHOP was commonly used. The most expeditious and efficacious method to reduce the size of the spleen and improve blood cell counts was splenectomy. Troussard et al reported a 5-year survival rate of 71% in 28 splenectomized patients [22]. While splenectomy rapidly corrects hypersplenism, thereby improving cytopenias, disease elsewhere is not treated. Splenectomy is associated with an operative mortality rate and a lifelong risk of infection and septicemia. Moreover, after splenectomy there is a postoperative increase in the tumor burden in the bone marrow. The success of Rituximab has revolutionized the treatment of NHL including SMZL, follicular lymphoma and diffuse large B-cell lymphoma. A study by Tsimberidou et al at the M.D. Anderson Cancer center showed an 88% response rate to rituximab monotherapy. Rituximab resulted in disappearance of a palpable spleen in 92% of the patients and was superior to splenectomy in normalizing absolute lymphocyte counts [23]. The use of rituximab in SMZL as an initial therapy is attractive since it can obviate the need for splenectomy with its attendant short- and long-term risks. Moreover, rituximab treatment does not compromise later splenectomy or chemotherapy if there is no response or in case of subsequent relapse [24]. A significant advantage in the rituximab plus chemotherapy group over the chemotherapy alone group was observed [23]. In our patient, the course of rituximab did shrink the spleen by 4 cm on image study with improved blood cell counts but not as reported, which may indicate the disease in the patient had poor response to rituximab. Splenectomy should be considered earlier in this case to decrease the tumor burden and improve clinical symptoms.
